# Acute Myocardial Infarction Risk in Patients with Coronary Artery Disease Doubled after Upper Gastrointestinal Tract Bleeding: A Nationwide Nested Case-Control Study

**DOI:** 10.1371/journal.pone.0142000

**Published:** 2015-11-03

**Authors:** Chia-Jung Wu, Hung-Jung Lin, Shih-Feng Weng, Chien-Chin Hsu, Jhi-Joung Wang, Shih-Bin Su, Chien-Cheng Huang, How-Ran Guo

**Affiliations:** 1 Department of Emergency Medicine, Chi-Mei Medical Center, Tainan, Taiwan; 2 Department of Emergency Medicine, Taipei Medical University, Taipei, Taiwan; 3 Department of Biotechnology, Southern Taiwan University of Science and Technology, Tainan, Taiwan; 4 Department of Medical Research, Chi-Mei Medical Center, Tainan, Taiwan; 5 Department of Healthcare Administration and Medical Informatics, Kaohsiung Medical University, Kaohsiung, Taiwan; 6 Department of Occupational Medicine, Chi-Mei Medical Center, Tainan, Taiwan; 7 Department of Leisure, Recreation and Tourism Management, Southern Taiwan University of Science and Technology, Tainan, Taiwan; 8 Department of Medical Research, Chi Mei Medical Center, Liouying, Tainan, Taiwan; 9 Department of Environmental and Occupational Health, College of Medicine, National Cheng Kung University, Tainan, Taiwan; 10 Department of Child Care and Education, Southern Taiwan University of Science and Technology, Tainan, Taiwan; 11 Department of Geriatrics and Geronotology, Chi-Mei Medical Center, Tainan, Taiwan; 12 Department of Occupational and Environmental Medicine, National Cheng Kung University Hospital, Tainan, Taiwan; University of Bologna, ITALY

## Abstract

Prior studies of upper gastrointestinal bleeding (UGIB) and acute myocardial infarction (AMI) are small, and long-term effects of UGIB on AMI have not been delineated. We investigated whether UGIB in patients diagnosed with coronary artery disease (CAD) increased their risk of subsequent AMI. This was a population-based, nested case-control study using Taiwan’s National Health Insurance Research Database. After propensity-score matching for age, gender, comorbidities, CAD date, and follow-up duration, we identified 1,677 new-onset CAD patients with AMI (AMI^[+]^) between 2001 and 2006 as the case group and 10,062 new-onset CAD patients without (AMI^[−]^) as the control group. Conditional logistic regression was used to examine the association between UGIB and AMI. Compared with UGIB^[−]^ patients, UGIB^[+]^ patients had twice the risk for subsequent AMI (adjusted odds ratio [AOR] = 2.08; 95% confidence interval [CI], 1.72–2.50). In the subgroup analysis for gender and age, UGIB^[+]^ women (AOR = 2.70; 95% CI, 2.03–3.57) and patients < 65 years old (AOR = 2.23; 95% CI, 1.56–3.18) had higher odds of an AMI. UGIB^[+]^ AMI^[+]^ patients used nonsignificantly less aspirin than did UGIB^[−]^ AMI^[+]^ patients (27.69% vs. 35.61%, respectively). UGIB increased the risk of subsequent AMI in CAD patients, especially in women and patients < 65. This suggests that physicians need to use earlier and more aggressive intervention to detect UGIB and prevent AMI in CAD patients.

## Introduction

Upper gastrointestinal bleeding (UGIB) is a common, costly, and potentially life-threatening disease [1]. It must be managed promptly and appropriately to prevent adverse outcomes [1]. Peptic ulcer bleeding is the most common type of UGIB, accounting for 31–67% of all cases, followed by erosive disease, variceal bleeding, esophagitis, malignancies, and Mallory-Weiss tear [1,2]. In the U.S., the annual rate of hospitalization for peptic ulcer disease and UGIB is estimated to be 165 per 100,000 in 1999—more than 300,000 hospitalizations per year, at a cost of $2.5 billion [3,4]. After antimicrobial drugs became available to eradicate H. pylori, the hospitalization rate for peptic ulcer disease decreased to 56.5/100,000 in 2005 (95% CI 54.6–58.3) [5]. Furthermore, despite advances in therapy, the mortality rate has remained unchanged at 7–10% [6]. This may be because today’s patients are older and have more comorbidities than did patients in the past [7].

UGIB can produce hypovolemia, hypotension, and diminished oxygen-carrying capacity, which causes myocardial ischemia and necrosis [8–11]. Prior studies of UGIB and AMI are small, and long-term effects of UGIB on AMI have not been delineated. In addition, all the previous studies focused on the simultaneous presentation of AMI and UGIB [8–11], and the subacute and long-term effects of UGIB on the subsequent risk of AMI in CAD patients were still unclear, even though the impact of UGIB is expected to be larger in CAD patients [9,10]. Therefore, we did a population-based, nested case-control study to investigate UGIB and the subsequent AMI risk in CAD patients.

## Materials and Methods

### Data sources

The Taiwan National Health Insurance (NHI) Program, a universal health care system that covers 99% of the country’s population of 23.3 million [12], has one of the world’s largest and most complete population-based healthcare claims datasets. The NHI Research Database (NHIRD) contains encrypted patient identification numbers, ICD-9-CM (International Classification of Diseases, Ninth Revision, Clinical Modification) codes for applied clinical diagnoses and procedures, details of prescribed drugs, dates of admission and discharge, and basic sociodemographic information, including gender and date of birth. All the expenses of CAD, UGIB, and AMI are covered by NHI.

The present study used a representative subset of the original NHIRD; this subset contains the claims information of 1,000,000 patients randomly selected from the NHI Registry of Beneficiaries 2000. The study was conducted according to the Declaration of Helsinki and was approved by the Chi-Mei Medical Center Institutional Review Board, which waived the need for informed consent because the dataset consists of nationwide, unidentifiable, secondary data released to the public for research. This waiver does not adversely affect the rights and welfare of the patients.

### Study population

**Fig 1:**
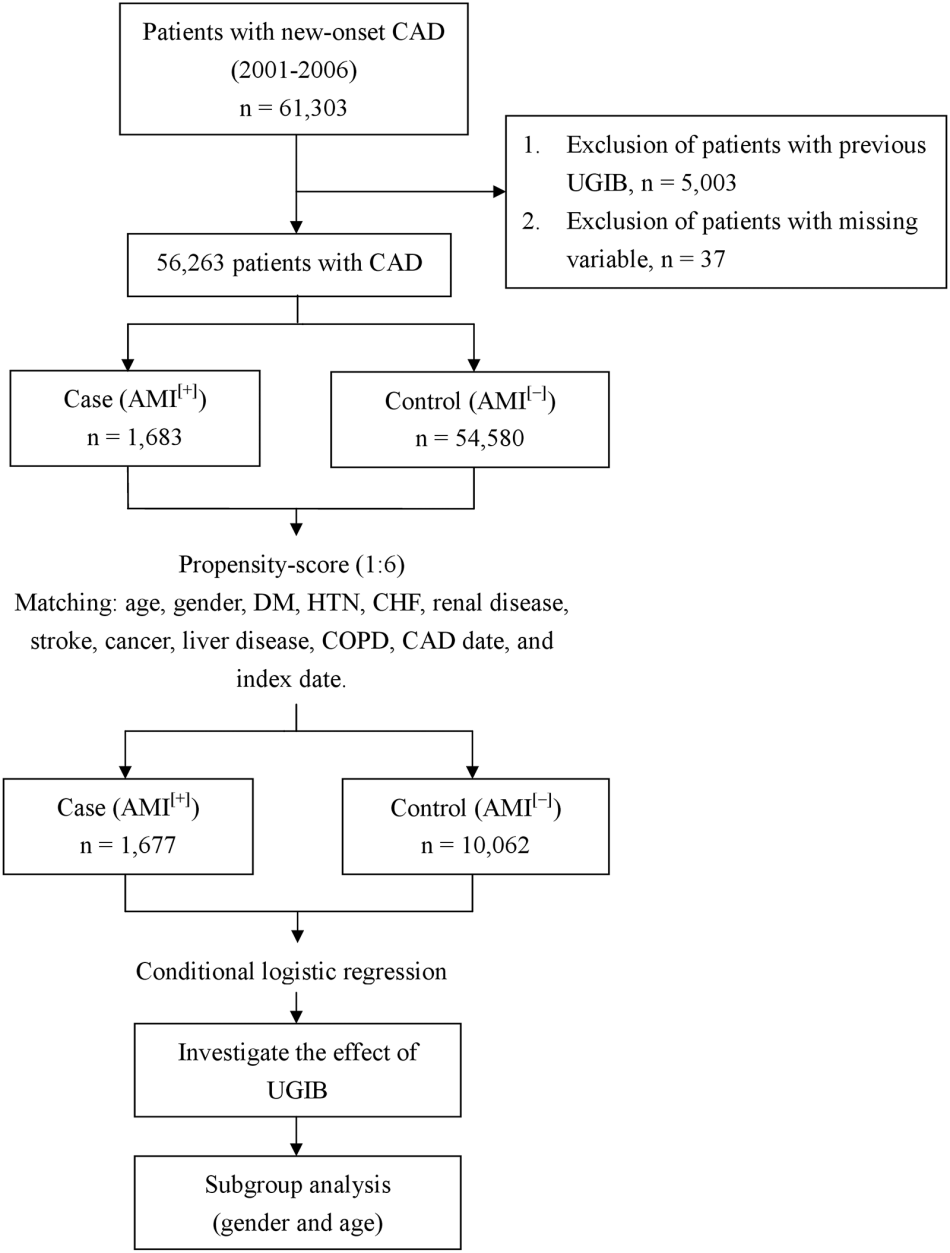
Flow diagram of the study. CAD, coronary artery disease; UGIB, upper gastrointestinal bleeding; DM, diabetes mellitus; HTN, hypertension; CHF, congestive heart failure; COPD, chronic obstructive pulmonary disease; AMI, acute myocardial infarction.

In this nested case control study, we identified all patients with new-onset CAD (ICD-9 codes 410–414.02) from 2001 to 2006 (n = 61,303) (Fig 1). The cohort entry date for each patient was defined as the date their first ambulatory or inpatient visit. Patients diagnosed with UGIB (ICD-9 codes 578.9, 531.0, 531.2, 531.4, 531.6, 532.0, 532.2, 532.4, 532.6, 533.0, 533.2, 533.4, 533.6, 534.0, 534.2, 534.4, and 534.6) before the study (n = 5,003) and patients with missing variables (n = 37) were excluded (Fig 1). Finally, 56,263 CAD patients without a history of UGIB (UGIB^[−]^) were enrolled (Fig 1). The cohort members were followed-up until they developed AMI, died, or withdrew from the NHI program, or until December 2011, whichever came first.

### AMI^[+]^ patients and AMI^[−]^ controls

The study outcome was AMI, which was defined as any admission for AMI based on ICD-9 codes 410.0–410.92. The date of the first AMI claim was defined as the date on which AMI was first clinically diagnosed (index date). The date of AMI onset was the same with the date of AMI claim. For each enrolled AMI patient, we randomly selected 6 propensity-score-matched controls from the new-onset AMI^[−]^ CAD^[+]^cohort. Controls and AMI^[+]^ patients were matched based on birth year, gender, comorbidities, CAD date (cohort entry date), and index date (Fig 1). Controls had the same index date as their corresponding matched patients. The comorbidities were diabetes mellitus (DM) (ICD-9 code 250), hypertension (HTN) (ICD-9 codes 401–405), congestive heart failure (CHF) (ICD-9 codes 428), renal disease (ICD-9 codes 582, 583, 585, 586, 588), stroke (ICD-9 codes 430–438), cancer (ICD-9 codes 140–208), liver disease (ICD-9 codes 571.2, 571.4, 571.5, 571.6, 456.0–456.2, 572.2–572.8), and chronic obstructive pulmonary disease (COPD) (ICD-9 codes 490–496, 500–505, 506.4). Propensity-score matching was used to reduce selection bias in our hypothesis because it can bundle many confounding covariates that may be present in an observational study with this number of variables. If there was no matching control, the AMI^[+]^ patient was excluded. Finally, 1,677 AMI^[+]^ patients and 10,062 AMI^[−]^ controls (1:6) from 2001 to 2006 were included (Fig 1).

### Exposure assessment

This nested case-control study compared the association between UGIB and AMI in new-onset CAD patients.

### Subgroup analyses

We analyzed the effect of a UGIB on developing AMI in new-onset CAD patients. Because both gender and age are well known risk factors for AMI, we performed stratified analyses to evaluate whether the possible effects of UGIB on AMI are different between the two genders and whether the effects are larger or smaller on the elderly. *Elderly* was defined as ≥ 65 years old (Fig 1).

### Statistical analysis

The significance of the differences in baseline characteristics and comorbid variables between the two groups was evaluated using Student’s *t* test for continuous variables and Pearson χ^2^ tests for categorical variables. The odds ratio (OR) and 95% confidence interval (CI) were calculated using conditional logistic regression adjusted for antiplatelet use to examine the association between UGIB and AMI. The comparison of antiplatelet use between UGIB^[+]^ and UGIB^[−]^ patients in the AMI^[+]^ group was done using a χ^2^ test. SAS 9.3.1 for Windows (SAS Institute, Inc, Cary, NC, USA) was used for all analyses. Significance was set at *P* < 0.05.

## Results

### Demographic data

After propensity-score matching, there were no significant differences in age, follow-up duration, gender, or comorbidities (Table 1). The mean age in the AMI^[+]^ group was 65.46 ± 12.68 years and in the AMI^[−]^ group was 65.63 ± 12.80 years. The combined mean follow-up duration for both groups was 3.30 ± 2.97 years (Table 1).

**Table 1:** Demographic characteristics of CAD patients. Data are presented as n (%) or means ± standard deviation. CAD, coronary artery disease; AMI, acute myocardial infarction; DM, diabetes mellitus; HTN, hypertension; CHF, congestive heart failure; COPD, chronic obstructive pulmonary disease.

	Before propensity-score matching (n = 56,263)			After propensity-score matching (n = 11,739)		
Characteristic	Case (AMI^[+]^)	Control (AMI^[−]^)	*P*-value	Case (AMI^[+]^)	Control (AMI^[−]^)	*P*-value
Number of patients	1,683	54,580		1,677	10,062	
Age (year)	65.47 ± 12.66	57.24 ± 15.67	<0.0001	65.46 ± 12.68	65.63 ± 12.80	0.6023
0–49	204 (12.12)	17,205 (31.52)	<0.0001	204 (12.16)	1,184 (11.77)	0.7893
50–64	553 (32.86)	18,805 (34.45)		552 (32.92)	3,266 (32.46)	
≥ 65	926 (55.02)	18,570 (34.02)		921 (54.92)	5,612 (55.77)	
Follow up duration (year)				3.30 ± 2.97	3.30 ± 2.97	1.0000
Gender						
Male	1,087 (64.59)	26,559 (48.66)	<0.0001	1,081 (64.46)	6,586 (65.45)	0.4286
Female	596 (35.41)	28,021 (51.34)	<0.0001	596 (35.54)	3,476 (34.55)	
Comorbidities						
DM	563 (33.45)	7,428 (13.61)	<0.0001	557 (33.21)	3,233 (32.13)	0.3797
HTN	852 (50.62)	18,473 (33.85)	<0.0001	846 (50.45)	5,039 (50.08)	0.7804
CHF	150 (8.91)	1,593 (2.92)	<0.0001	144 (8.59)	805 (8.00)	0.4148
Renal disease	138 (8.20)	1,555 (2.85)	<0.0001	132 (7.87)	701 (6.97)	0.1817
Stroke	229 (13.61)	3,573 (6.55)	<0.0001	227 (13.54)	1,339 (13.31)	0.7988
Cancer	43 (2.55)	1,264 (2.32)	0.5213	43 (2.56)	256 (2.54)	0.9618
Liver disease	83 (4.93)	3,522 (6.45)	0.0121	83 (4.95)	467 (4.64)	0.5804
COPD	199 (11.82)	4,343 (7.96)	<0.0001	197 (11.75)	1,154 (11.47)	0.7409

### UGIB and subsequent risk of AMI

Of the 1,677 AMI^[+]^ patients, 242 (14.43%) had UGIB before AMI (Table 2). In the AMI^[−]^ control group, 890 of 10,062 patients (8.85%) had UGIB before the index date (Table 2). The adjusted OR (AOR) for AMI^[+]^ patients with UGIB was 2.08 (95% CI, 1.72–2.50).

**Table 2:** UGIB and subsequent risk of AMI in CAD patients. UGIB, upper gastrointestinal bleeding; AMI, acute myocardial infarction; CAD, coronary artery disease; OR, odds ratio. Adjusted by antiplatelet use.

Variable	Case (AMI^[+]^)	Control (AMI^[−]^)	Adjusted OR	*P*-value
UGIB				
Yes, n (%)	242 (14.43)	890 (8.85)	2.08 (1.72–2.50)	<0.0001
No, n (%)	1,435 (85.57)	9,172 (91.15)	1.00	

### Subgroup analyses stratified by gender and age

In the subgroup analysis for gender, the AOR for AMI^[+]^ patients who had UGIB was 1.65 (95% CI, 1.28–2.13) for males and 2.70 (95% CI, 2.03–3.57) for females (Table 3). In the subgroup analysis for age, the AOR for AMI^[+]^ patients who had UGIB was 2.23 (95% CI, 1.56–3.18) for patients < 65 and 2.01 (95% CI, 1.59–2.52) for patients ≥ 65 (Table 4).

**Table 3:** Gender subgroup of UGIB and subsequent risk of AMI in CAD patients. UGIB, upper gastrointestinal bleeding; AMI, acute myocardial infarction; CAD, coronary artery disease; OR, odds ratio. Adjusted by antiplatelet use.

Male				
Variable	Case (AMI^[+]^)	Control (AMI^[−]^)	Adjusted OR	*P*-value
UGIB				
Yes, n (%)	125 (11.56)	563 (8.55)	1.65 (1.28–2.13)	0.0001
No, n (%)	956 (88.44)	6,023 (91.45)	1.00	
Female				
Variable	Case (AMI^[+]^)	Control (AMI^[−]^)	Adjusted OR	*P*-value
UGIB				
Yes, n (%)	117 (19.63)	327 (9.41)	2.70 (2.03–3.57)	<0.0001
No, n (%)	479 (80.37)	3,149 (90.59)	1.00	

**Table 4:** Age subgroup of UGIB and subsequent risk of AMI in CAD patients. UGIB, upper gastrointestinal bleeding; AMI, acute myocardial infarction; CAD, coronary artery disease; OR, odds ratio. Adjusted by antiplatelet use.

Non-elderly (0 < 65 years)				
Variable	Case (AMI^[+]^)	Control (AMI^[−]^)	Adjusted OR	*P*-value
UGIB				
Yes, n (%)	85 (11.24)	298 (6.70)	2.23 (1.56–3.18)	<0.0001
No, n (%)	671 (88.76)	4,152 (93.30)	1.00	
Elderly (≥ 65 years)				
Variable	Case (AMI^[+]^)	Control (AMI^[−]^)	Adjusted OR	*P*-value
UGIB				
Yes, n (%)	157 (17.05)	592 (10.55)	2.01 (1.59–2.52)	<0.0001
No, n (%)	764 (82.95)	5,020 (89.45)	1.00	

### Antiplatelet use in the AMI^[+]^ group

AMI^[+]^ patients who were also UGIB^[+]^ took less aspirin (27.69% vs. 35.61%), more clopidogrel (6.61% vs. 5.85%), and less aspirin + clopidogrel (23.97% vs. 24.32%) than did AMI^[+]^ patients who were UGIB^[−]^ (Table 5). However, the difference was not significant (*P* = 0.0629).

**Table 5:** Antiplatelet use in the AMI^[+]^ group.

Medications	UGIB^[+]^ n = 242	UGIB^[−]^ n = 1,435	*P*-value
Aspirin	67 (27.69)	511 (35.61)	0.0629
Clopidogrel	16 (6.61)	84 (5.85)	
Aspirin + Clopidogrel	58 (23.97)	349 (24.32)	
None	101 (41.74)	491 (34.22)	

### Proton pump inhibitors and H_2_ receptor blockers use in the UGIB^[+]^ group

UGIB^[+]^ patients who were also AMI^[+]^ took more proton pump inhibitors (71.07% vs. 49.10%, *P* <0.0001) and more H_2_ receptor blockers (6.61% vs. 5.85%, *P* = 0.0011) than did UGIB^[+]^ patients who were AMI^[−]^ (Table 6).

**Table 6:** Proton pump inhibitors and H_2_ receptor blockers use in the UGIB^[+]^ group.

Medications	AMI^[+]^ n = 242	AMI^[−]^ n = 890	*P*-value
Proton pump inhibitors	172 (71.07)	437 (49.10)	<0.0001
H_2_ receptor blockers	122 (50.41)	345 (38.76)	0.0011

## Discussion

We found that patients with CAD and then UGIB, especially women and those < 65 years old, had twice the risk of developing an AMI than did CAD patients without UGIB. To prevent AMI and subsequent mortality and morbidity, early referral of CAD patients with UGIB for additional evaluation and treatment may be needed.

Other studies have reported that the prevalence of AMI in patients with UGIB ranges from 1% to 14% [8–11]. There is no general agreement about risk stratification of AMI in patients with UGIB. Some studies [8–11,13,14] suggest that those with a greater number of coronary risk factors, a history of CAD, lower blood pressure on admission, older age, severe illnesses, or lower hemoglobulin have a greater risk of AMI.

There are two scenarios between UGIB and AMI. The first one is that UGIB predisposes a patient to develop AMI, and the second is that AMI predisposes a patient to develop UGIB. Cappell (2000) suggested that when UGIB precipitates an AMI, it tends to be acute and massive, whereas UGIB precipitated after an AMI has been treated tends to be self-limited and often resolves with the reversal of underlying coagulopathy [15]. We found that UGIB has a continuing potential to induce a subsequent AMI in CAD patients. This may be because of (i) anemia and hypoperfusion: AMI secondary to ischemia may be due to a decrease in the body’s oxygen supply. Bellotto (2005) showed that hemoglobin ≤ 8.2 g/dL was a significant risk factor for myocardial necrosis [8]. Activating the sympathetic nervous system can increase demand for myocardial oxygen and worsen ischemia; or (ii) discontinuing of antiplatelet use: antiplatelet drug therapy, such as aspirin and clopidogrel, should be continued to reduce the risk of plaque rupture and recurrent AMI. In the present study, UGIB^[+]^ AMI^[+]^ patients used less aspirin than did UGIB^[+]^ AMI^[−]^ patients. Aspirin was always discontinued after UGIB because aspirin doubles the risk of bleeding ulcers, even at doses as low as 75 mg daily [16]. Aspirin is first-line, because of its low cost and comparable efficacy, and clopidogrel is reserved for patients who cannot tolerate aspirin [17]. The combination of clopidogrel and aspirin may reduce the risk of cardiovascular events, but it raises the risk of hemorrhage [17]. In total, 64.7% AMI^[+]^ patients have antiplatelet therapy (aspirin, clopidogrel, or both), which was a relative low percentage. There was no report about the compliance of antiplatelet therapy in patients with CAD in Taiwan. However, a study about antihypertensive medication compliance in Taiwan reported only 53% of the patients had high compliance with antihypertensive medication [18], which may be due to the characteristics of this population. The present study also showed UGIB^[+]^ AMI^[+]^ patients took more proton pump inhibitors and more H_2_ receptor blockers than did UGIB^[+]^ AMI^[−]^ patients, which suggested that both of these two drugs may not reduce the AMI risk in UGIB^[+]^ patients.

In the subgroup analysis, we found that women and patients < 65 years old had twice the risk of developing an AMI than did CAD patients without UGIB, which has never been reported before. Other studies [8–11,13,14] reported that older age is a risk factor and simultaneous UGIB and AMI occurred most often in men. Although our data do not give us sufficient information to explain this apparent discrepancy, we hypothesize that women and patients < 65 have fewer comorbidities than do men and the elderly; therefore, UGIB is the most likely factor that contributes to AMI.

This study has some limitations. First, the comorbidities relied on the claims data and ICD-9-CM diagnosis codes, which may have resulted in disease misclassification. However, many studies have validated that ICD-9-CM diagnosis codes of NHIRD have a high sensitivity and specificity for the actual diagnosis [19–21]. Second, the NHIRD contains no information on the severity of UGIB and AMI; therefore, we were unable to evaluate the severity association between them. Third, some important laboratory data, such as hemoglobin concentrations, and drugs, such as NSAIDs (nonsteroid anti-inflammatory drugs) and SSRI (selective serotonin reuptake inhibitors), were not available in the NHIRD, therefore, we could not interpret the available results fully and adjust for these variables as contributing factors in this study. Further study about this issue is warranted. Fourth, whereas we observed a temporal association between UGIB and AMI, we were unable to provide the risk estimates of AMI within certain periods of time (e.g. 1, 6, or 12 months of the bleed) and what percentage of patients discontinued aspirin or changed antiplatelet therapy which may affects subsequent AMI. However, this study showed that AMI^[+]^ patients who were also UGIB^[+]^ took less aspirin and less aspirin + clopidogrel than did AMI^[+]^ patients who were UGIB^[−]^, which was an indirect evidence of discontinue for aspirin. In addition, while diminished oxygen carrying capacity following UGIB is a plausible explanation, the presentation of AMI in such cases would be acute rather than subacute. A cohort study that recruits a group of CAD patients who have UGIB and follows them over time for AMI episodes after the UGIB including discontinue of aspirin is needed to obtain such estimates. Fifth, we did not identify the origin of UGIB and recurrent UGIB. However, the aim of this study was to evaluate whether CAD patients have a higher risk of developing AMI after UGIB, and therefore further studies that identify the origin of UGIB and recurrent UGIB are warranted for developing more specific prevention strategies. Sixth, although our database was national, our findings may not be generalizable to similar cohorts in other nations.

## Conclusions

This is the first nationwide population-based, nested case-control study to clarify that CAD patients, especially women and those < 65 years old, have twice the risk of developing an AMI after UGIB than do CAD patients who do not have UGIB. Therefore, early referral of CAD patients who develop UGIB for additional evaluation and treatment, including a modification of antiplatelet therapy, is needed to lower the risk of developing AMI.
